# Classification of GABAergic interneurons by leading neuroscientists

**DOI:** 10.1038/s41597-019-0246-8

**Published:** 2019-10-22

**Authors:** Bojan Mihaljević, Ruth Benavides-Piccione, Concha Bielza, Pedro Larrañaga, Javier DeFelipe

**Affiliations:** 1 Computational Intelligence Group, Departamento de Inteligencia Artificial, Universidad Politécnica de Madrid, Boadilla del Monte, 28660 Spain; 2 Laboratorio Cajal de Circuitos Corticales, Universidad Politécnica de Madrid and Instituto Cajal (CSIC), Pozuelo de Alarcón, 28223 Spain

**Keywords:** Neural circuits, Cellular neuroscience

## Abstract

There is currently no unique catalog of cortical GABAergic interneuron types. In 2013, we asked 48 leading neuroscientists to classify 320 interneurons by inspecting images of their morphology. That study was the first to quantify the degree of agreement among neuroscientists in morphology-based interneuron classification, showing high agreement for the chandelier and Martinotti types, yet low agreement for most of the remaining types considered. Here we present the dataset containing the classification choices by the neuroscientists according to interneuron type as well as to five prominent morphological features. These data can be used as crisp or soft training labels for learning supervised machine learning interneuron classifiers, while further analyses can try to pinpoint anatomical characteristics that make an interneuron especially difficult or especially easy to classify.

## Abstract (summary)

**Table:** 

Measurement(s)
Technology Type(s)
Factor Type(s)
Sample Characteristic - Environment

Machine-accessible metadata file describing the reported data: 10.6084/m9.figshare.9948803

## Background & Summary

There is currently no unique catalog of cortical GABAergic interneuron types^1^. Forming such a catalog is a major goal in neuroscience and is currently pursued by, among others, the Human Brain Project, the Allen Institute and the BRAIN initiative^2,3^. While high-throughput data generation may enable a fully data-driven classification of interneurons in near future, by clustering^4,5^ molecular, morphological, and electrophysiological features, researchers currently use established morphological types such as chandelier, Martinotti, neurogliaform, and basket^6–10^.

**Fig. 1:**
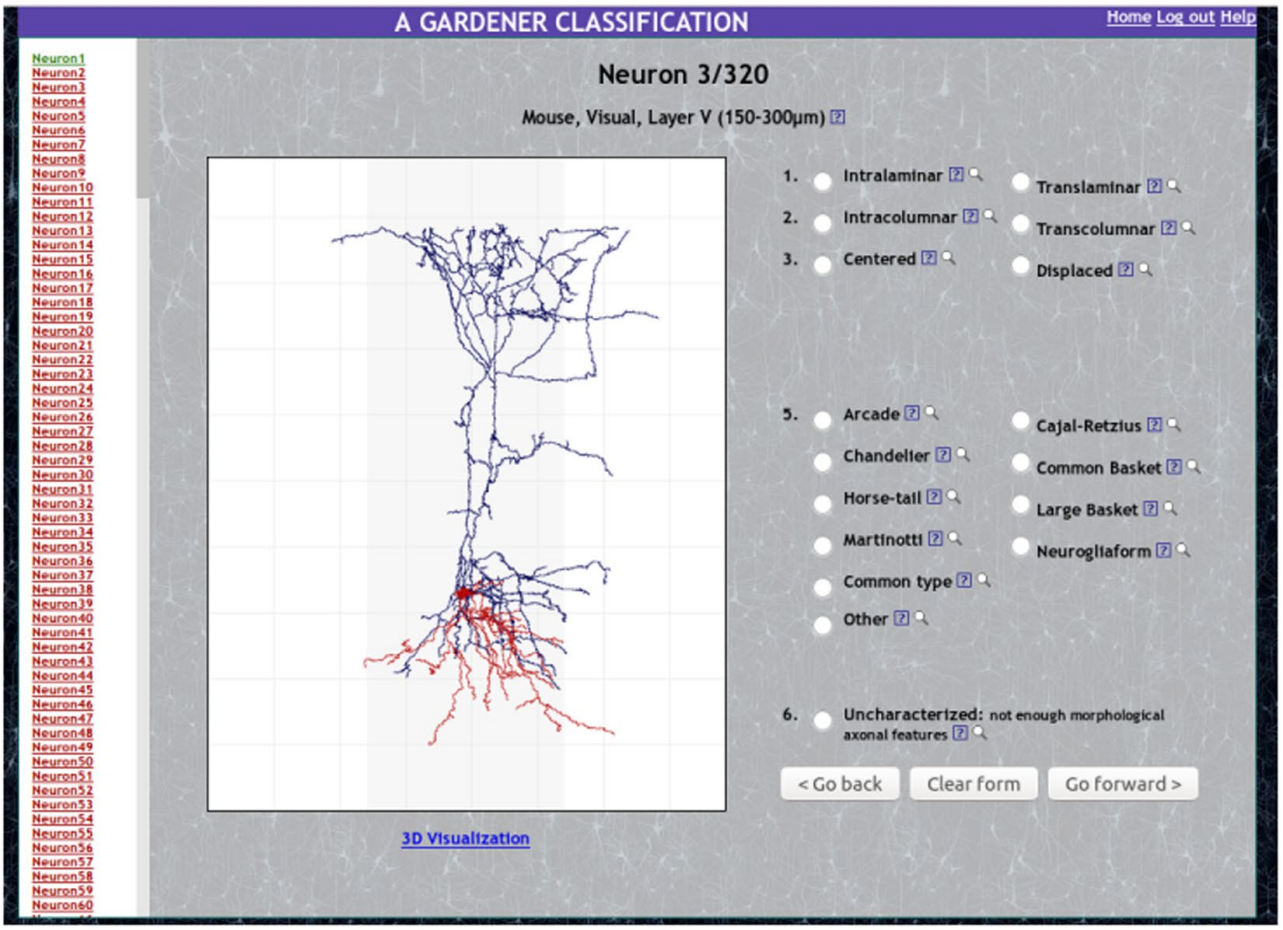
The web application used to gather the neuroscientists’ classification choices for cortical GABAergic interneurons.

In 2013, we asked 48 leading neuroscientists to classify 320 interneurons by inspecting 2D and 3D images of their morphology (ref.^7^ see Fig. 1). This landmark study was the first to quantify the degree of agreement among neuroscientists in morphology-based interneuron classification, showing high agreement for the chandelier and Martinotti types, yet low for most of the remaining types considered such as, for example, the large basket type. In addition to interneuron type, the neuroscientists also classified the cells according to prominent morphological features, such as whether an axon was intra- or trans-laminar.

In this report, we present the data collected by^7^, namely the labeling choices made by the 48 neuroscientists. We also provide the input that the neuroscientists had when classifying the cells: the 2D morphology images that they looked at, cell metadata they were shown, and the definitions of interneuron types and morphological features of interest. For 241 of the cells we provide their morphology reconstructions as well as their Neuromorpho.org^11^ ids, so that one can obtain additional metadata. We report a posteriori data curation, such as identifying ten cells that were shown to the annotators rotated upside-down. We also provide an R package with utility functions for analyzing the data.

Besides enabling one to reproduce the study by^7^, these data allow for further analyses. They have been used to assign class labels for supervised^7,12,13^ and semi-supervised^14^ classification of interneurons, to cluster neuroscientists according to their classification choices^15^, to quantify neuroscientists’ accuracy when identifying Martinotti cells from morphology images and use it as a baseline for assessing supervised classifiers^16^, as well as to contrast the classification choices of these 48 neuroscientists to those from a particular research group^16^.

Combining the here provided classification choices can give a crisp or soft (i.e., probabilistic) estimate of the type of these 320 neurons, insofar as the type can be accurately determined from an image of the morphology along with basic metadata. Since the classification choices come from many leading neuroscientists, these combined estimates are objective, i.e., they represent a consensus among experts from different laboratories. Assessing and accounting for the accuracy of the annotators (e.g.^17–19^) might give better estimates of the type than if assuming that they are equally accurate, as we have done in our previous work. Further analyses may consider per-species or laminar differences in inter-neuroscientist agreement, or can try to pinpoint characteristics that distinguish interneurons that are especially difficult to classify from those that clearly belong to a given type. For example, while there was little inter-neuroscientist agreement on the large basket type, some cells were clearly members of this type as they were labeled as large basket cells by a majority of the neuroscientists.

## Methods

All data were collected by^7^ and data acquisition is described in their paper. Here we provide a self-contained description of the neurons, the classification experiment, and curation, so that the data can be used by referring to this publication only.

### Classification web application

Each neuroscientist used the web application shown in Fig. 1 to classify interneurons. In addition to 2D images, which were available for all interneurons, 3D visualization was provided for 241 of the cells, allowing the neuroscientists to rotate and zoom the morphologies. The cell’s brain area, cortical layer and estimated layer thickness were stated when available, as well as the species of the animal. A help page provided definitions of neuronal types and categories. The web application that the neuroscientists used to classify the cells can be accessed at http://cajalbbp.es/gardenerclassification/. Throughout the paper, we use the term ‘annotators’ to refer to the 48 neuroscientists that participated in the study, *annotating* (i.e., classifying) the selected cells.

### Interneuron selection

Reference^7^ asked the neuroscientists to classify 320 cortical GABAergic interneurons. The authors downloaded 241 of these cells from Neuromorpho.org^11^, and obtained pictures of the remaining 79 interneurons by scanning images from scientific publications (our dataset includes the 2D images of all 320 cells; see below). The cells come from different cortical areas and layers of the mouse, rat, rabbit, cat, monkey and human. The authors obtained cell metadata from Neuromorpho.org and the scanned papers. The layer of the soma was unknown for 30 cells from Neuromorpho.org.

### Classification scheme

**Fig. 2:**
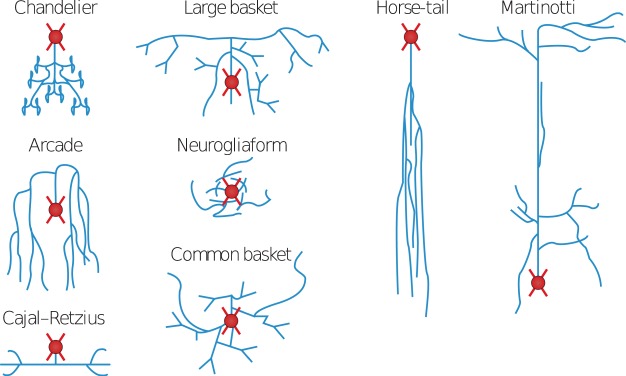
Interneuron types in the gardener’s scheme. Figure from^7^. Reprinted by permission from Nature Reviews Neuroscience.

Reference^7^ proposed a classification scheme based mainly on patterns of axonal arborization. The scheme contemplates ten interneuron types (see Fig. 2): arcade, Cajal-Retzius, chandelier, common basket, common type, horse-tail, large basket, Martinotti, neurogliaform, and other. Other is meant to be chosen when the neuroscientist finds none of the remaining nine types adequate and prefers to use an alternative name. Full definitions of the types are provided in the data (see below).

In addition to interneuron type, the classification scheme contemplates five high-level morphological features, such as whether or not the axon is restricted to the layer that contains the soma. These features, termed F1, F2, F3, F4, and F6 (F5 is the previously discussed interneuron type) have the following categories: (F1) intralaminar and translaminar; (F2) intracolumnar and transcolumnar; (F3) centered and displaced; (F4) ascending, descending, and both; (F6) characterized and uncharacterized. The uncharacterized category of F6 means that a cell’s reconstruction is not good enough to reliably classify it. When labeling a cell as uncharacterized in feature F6, the neuroscientist cannot annotate it according to any of the remaining five features, F1-F5. F4 is only applicable for cells that are labeled as translaminar and displaced in F1 and F3, respectively. Full definitions of features F1-F6 are provided in the data.

### Annotation

A total of 48 neuroscientists participated in the study. 42 of them fully classified all 320 neurons, thus providing 42 × 320 × 6 = 80,640 labels. Six neuroscientists classified a subset of the 320 cells (150, on average), with four of them failing to assign labels to all of F1-F6 for some cells, thus providing a total of another 4,452 labels.

### A posteriori curation

We found, by comparing 97 of our Neuromorpho.org reconstructions to reconstructions that we got directly from the original laboratory, that ten cells were rotated upside-down at Neuromorpho.org and were thus displayed as such to the neuroscientists. We report which cells were shown with their morphologies upside-down (see below).

We provide the additional metadata that we obtained from Neuromorpho.org: the original cell type (the ‘Secondary Cell Class’ attribute at Neuromorpho.org), the reconstructing laboratory (‘Archive’), and the name of a reference article. We also provide morphology reconstruction files that we downloaded from Neuromorpho.org for 241 cells.

## Data Records

All data are hosted at figshare^20^. The neuroscientists’ annotations are stored in annotations.csv (see example in Table 1). The numbering for annotators 1 to 42 follows that in^7^ –e.g., the ids can be matched to those used, for example, in their Fig. 19 of Ref.^7^ –while ids larger than 42 correspond to neuroscientists that labeled less than 320 interneurons and were thus not considered in^7^. The neurons also maintain the ids, ranging from 1 to 320, used in the original classification web application and by^7^. The ‘complete’ column in annotations.csv indicates where the neuron was completely or partially labeled by a particular neuroscientist. In all files, ‘None’ means that an entry was not applicable to a given interneuron (e.g., F4 for annotator 1, cell 1, in Table 1), while an empty entry (e.g., F2 for annotator 45, cell 1, in Table 1) denotes a missing value.

**Table 1:** Annotation of interneurons 1, 79 and 80 by neuroscientists 1, 16, and 45.

Annotator	Neuron	F1	F2	F3	F4	F5	F6	Other
1	1	intralaminar	intracolumnar	centered	None	neurogliaform	characterized	None
16	1	intralaminar	intracolumnar	centered	None	neurogliaform	characterized	None
43	203	translaminar	intracolumnar	centered	None	common basket	characterized	None
1	79	translaminar	intracolumnar	displaced	both	other	characterized	columnar basket
16	79	translaminar	intracolumnar	displaced	ascending	Martinotti	characterized	None
43	281	translaminar	intracolumnar	displaced	ascending	horse-tail	characterized	None
1	80	None	None	None	None	None	uncharacterized	None
16	80	intralaminar	intracolumnar	centered	None	common type	characterized	None
43	282	translaminar	intracolumnar	displaced	descending	horse-tail	characterized	None

Definitions for alternative interneuron type names provided by the neuroscientists, which they used in column ‘other’ of Table 1, are stored in alternative-types.csv (see Table 2). There are a total of 269 alternative type names, of which 251 are unique (241 are unique if we remove the ‘?’ symbol from type names). 163 types have a name but no definition (e.g., see annotator 14 in Table 2) while one type, assigned to 38 neurons by a single neuroscientist, also lacks a name (last row in Table 2). The annotators.csv file lists the names and affiliations in 2013 of the 48 neuroscientists that participated in the study. The names are given in an alphabetic order, unrelated to the annotator ids used in annotations.csv.

**Table 2:** Examples of alternative type names and definitions provided by the neuroscientists. An alternative type is uniquely defined by the annotator id along with its name.

Annotator	Type	Definition
1	columnar basket	This term is not new, I believe. In my view, these cells have a pattern of…
4	bitufted	I made a mistake. The bitufted cell should be classified as bipolar horseta…
4	ascending horsetail	Cells with horsetail-shaped ascending axons…
4	bipolar horsetail	Cells with horsetail-shaped ascending and descending axons…
7	deep layer inhibitor	neuron with an axonal domain that targets preferentially deep cortical laye…
7	double bouquet	neuron with an axonal domain that targets both deep and superficial layers…
14	narrow arbor cell	
18	bitufted?	see above…
23	bitufted	
43		

Basic cell metadata, with corrected brain area information, is in file metadata.csv (see Table 3). Column ‘neuromorpho.name’ corresponds to the ‘Neuron name’ attribute at Neuromorpho.org. Cells that were rotated upside-down are marked with TRUE in the ‘rotated’ column, while the original type, as reported at Neuromorpho.org, is provided in column ‘original.type’. We provide the name of the original paper when the paper is reported at Neuromorpho.org (not shown in Table 3).

**Table 3:** Partial (eight out of nine columns in metadata.csv) metadata for six cells.

Neuron	Neuromorpho.name	Species	Area	Layer	Rotated	Original.type
1	None	Monkey	Visual	IV	FALSE	
2	2001-11-09-B-L23-dendax	Rat	Somatosensory	II/III	FALSE	Not reported
3	020801-2-ST	Mouse	Visual	V	FALSE	Somatostatin
						containing cell
6	None	Monkey	Visual	IV	FALSE	
29	C170998D-I4	Rat	Somatosensory	II/III	TRUE	Basket cell
35	C170897A-I1	Rat	Somatosensory	IV	TRUE	Basket cell

The neuromorpho-swcs folder contains morphology reconstructions for 241 interneurons that we downloaded from Neuromorpho.org in August, 2019. The reconstructions correspond to standardized morphology files from Neuromorpho.org, encoded in the SWC format. Each reconstruction filename is given by the ‘neuromorpho.name’ of the neuron followed by ‘CNG.swc‘ (e.g., C170998D-I4.CNG.swc for neuron with ‘neuromorpho.name’ C170998D-I4). The urls file contains the Neuromorpho.org URLs that we downloaded the reconstructions from, so users can easily re-download them. While unlikely, some reconstructions may be affected by future curation efforts at Neuromorpho.org. We thus recommend users to consider re-downloading the reconstructions from Neuromorpho.org instead of using the reconstructions provided in neuromorpho-swcs.

The annotation-input folder contains input given to the neuroscientists during the classification experiment: the 2D images of neurons (in the images subfolder), exact definitions of the types and features (instructions.pdf), cells’ metadata as it was shown to them in metadata.csv, and estimates of cortical layer thickness per species and brain area (areamap.txt).

## Technical Validation

Most of the neuronal reconstructions were thorough and were therefore considered as characterizable by the neuroscientists. Shortcomings of the study include the upside-down display of ten cells, unknown or unreported layer of the soma for 30 cells, and a mistakenly reported brain area for 43 cells.

The quality of the classification labels stems from the fact that they come from a diverse set of 48 leading neuroscientists. We did not control for labeling mistakes, as one could do by, for example, showing an interneuron twice to an annotator and comparing the two assigned labels. As a surrogate measure of consistency, we compared the interneuron type labels by four neuroscientists to their own classification of the same cells in their previous papers. The four neuroscientists were annotators that were co-authors of papers associated with the neurons that we obtained from Neuromorpho.org. We restricted our attention to cells whose original label (i.e., the ‘Secondary Cell Class’ attribute at Neuromorpho.org) was either chandelier, basket, Martinotti, or neurogliaform. We disregarded cells that were shown rotated upside down, thus obtaining 104 label pairs to compare. When matching the provided labels with the original ones, we considered arcade, common basket and large basket as matching the Neuromorpho.org basket type; we include the arcade type here because it is often regarded as an alternative name for the nest basket type (see, e.g., Table 1 in^8^).

The labels that the four neuroscientists assigned in our study matched their original labels 86% of the time (in 91 out of 104 cells). We consider that this number indicates high labeling consistency by these four annotators, given the differences between our experimental setting and the setting in which they provided the original labels. For example, a major difference is that, when performing the original classification, authors are likely to have had access to additional data such as physiological or molecular characteristics of the cell, or low-level morphological features such as the distribution of boutons.

Note that even imperfect classification by many annotators can provide very accurate results when combining their output. Namely, statistics tells us that that combining the output of many diverse predictors that are individually better than random guessing tends to produce very accurate predictions^21^. Thus, as long as our group of annotators is diverse enough, and they are individually better than random guessing–which is to be expected given their expertise–combining their labels by simple majority will tend to provide good labels. More sophisticated combining methods^17–19^ might identify and account for any systematic mistakes among annotators (e.g., some might be more familiar with certain cell types than with others).

## Usage Notes

The data can be downloaded from figshare^20^. Since the data consist of comma-separated values and plain text files, they can be easily handled with standard data analysis software. We also provide the gardenr R package with a utility functions to access the data and examples of analyses. The package can be installed from Github with devtools::install_github(‘ComputationalIntelligenceGroup/gardenr’). It loads the data into R and shows how to combine data from three csv files–namely, on annotations, metadata and alternative types data–to perform meaningful analyses. Some utility functions for combining data are provided, while advanced filtering and summarization are easy to perform with the tidyverse packages.

## Data Availability

The code of the web application (Fig. 1) that^7^ used to collect the neuroscientists’ inputs is not publicly available.
